# Dietary Effects of Lipid and Protein Levels on Growth, Feed Utilization, Lipid Metabolism, and Antioxidant Capacity of Triploid Rainbow Trout (*Oncorhynchus mykiss*)

**DOI:** 10.1155/2023/8325440

**Published:** 2023-08-25

**Authors:** Yun-Feng Chen, Kai-Lin Cao, Hong-Fei Huang, Xiao-Qin Li, Xiang-Jun Leng

**Affiliations:** ^1^National Demonstration Center for Experimental Fisheries Science Education, Shanghai Ocean University, Shanghai 201306, China; ^2^Centre for Research on Environmental Ecology and Fish Nutrition (CREEFN) of the Ministry of Agriculture, Shanghai Ocean University, Shanghai 201306, China; ^3^Shanghai Collaborative Innovation for Aquatic Animal Genetics and Breeding, Shanghai Ocean University, Shanghai 201306, China

## Abstract

This study investigated the dietary effects of lipid and protein levels on growth performance, feed utilization, body composition, lipid metabolism, and antioxidant capacity of triploid rainbow trout, *Oncorhynchus mykiss*. A 3 × 2 two-factor design was conducted with three crude lipid levels of 4%, 9%, and 14% (L4, L9, and L14) and two crude protein levels of 44%, 49% (P44, P49). Therefore, a total of six diets were prepared as P44/L4, P44/L9, P44/L14, P49/L4, P49/L9, and P49/L14. Triploid rainbow trout (initial body weight 65.0 ± 0.1 g) were fed one of the six diets for 80 days. The results showed that weight gain (WG), protein retention (PR), and protein efficiency rate (PER) significantly increased with increasing the dietary lipid level at the same crude protein level, while feed conversion ratio (FCR) and hepatosomatic index significantly decreased (*P* < 0.05). At the same lipid level, there was no difference in WG, FCR, PR, PER between 44% and 49% crude protein group (*P* > 0.05). The P49/L14 group had the highest WG (374.6%) and lowest FCR (1.25), while P44/L14 group had the highest PER (1.80) and PR (25.06%) with similar WG and FCR to P49/L14 group. The crude lipid contents in whole fish were significantly higher in the L14 group than those in the L4 and L9 groups (*P* < 0.05). Muscle *n*-3 PUFAs, *n*-6 PUFAs, and PUFAs levels were positively correlated with dietary lipid level, while *n*-6 PUFAs was negatively correlated with dietary protein level. Dietary protein, dietary lipid, and their interaction significantly affected hepatic malondialdehyde (MDA) content, aspartate aminotransferase, lipase (LPS), and fatty acid synthase (FAS) activities (*P* < 0.05). In both P44 and P49 groups, LPS and FAS activities increased with increasing the dietary lipid level. MDA content significantly decreased in the P44 group and increased in the P49 group with increasing the dietary lipid level (*P* < 0.05). As dietary protein level increased, serum total cholesterol level increased, while hepatic phosphoenolpyruvate carboxykinase activity decreased. With increasing the dietary lipid level, total superoxide dismutase, catalase, total nitric oxide synthase, and fructose-1,6-bisphosphatase activities showed an increasing trend, while the opposite was true for alanine aminotransferase activity. In conclusion, based on growth performance and feed utilization, dietary protein level of 44% and dietary lipid level of 14% (measured value, 43.71% and 13.62%) were suggested for young triploid rainbow trout.

## 1. Introduction

Protein is one of the most important nutrients of the animal organism. As an important nutrient and energy source, protein has unique physiological functions and metabolic effects. For example, protein can be used as raw materials for the synthesis of enzymes, hormones, and other metabolites [1]. In addition, it is important to have the appropriate level of protein in the fish feed. In Wuchang bream (*Megalobrama amblycephala*), the weight gain (WG) and muscle protein content were significantly increased, while the feed conversion ratio (FCR) and muscle lipid content were significantly reduced as dietary protein level increased from 30% to 34% [2]. However, high dietary protein level increased the feed costs [3] and ammonia emissions [4]. Güroy et al. [4] found that increasing dietary protein level from 35% to 50% significantly increased the ammonia emissions in yellow tail cichlid (*Pseudotropheus acei*).

Lipid is a major energy-producing substance, and it is also the source of essential fatty acids [5]. Previous studies have reported that dietary lipid enhanced the absorption and transportation of fat-soluble nutrients [6], affecting the processing quality of feed and the shelf life of fish products [7]. Excessive dietary lipid level may exacerbate the fat deposition in the body and produce fatty fish [8]. In addition, dietary lipid level also affects the muscle quality of fish including the lipid deposition in brown trout (*Salmo trutta*) [9] and flesh color and flavor in rainbow trout (*Oncorhynchus mykiss*) [10, 11]. Different from lipid and carbohydrate, excess dietary protein level would be used for intermediate metabolism in the form of energy, or converted to glucose or lipid retention [12]. In blunt snout bream [3], Atlantic salmon (*Salmo salar*) [13], and rainbow trout [14], lipid showed a protein-sparing effect. In addition, there may be an interaction between dietary protein and lipid [3]. For example, nitrogen retention efficiency of largemouth bass (*Micropterus salmoides*) was significantly affected by dietary protein and lipid interaction [15]. WG, hepatosomatic index (HSI), and viscerosomatic index (VSI) of red-spotted grouper (*Epinephelus akaara*) were also significantly affected by dietary protein and lipid interaction [16]. Therefore, from the perspective of achieving optimal growth, reducing feed costs, and improving the culture environment, it is important to investigate the protein and lipid requirement as well as their interaction effects on cultured fishes.

Rainbow trout is one of the most appropriate fish for cold water farming [17] which has an annual global production over 848,000 tons [18]. The traditionally farmed rainbow trout is diploid, but in recent years, triploid rainbow trout have become an important farmed fish in China with annual production of >30,000 tons [19]. Triploid rainbow trout have three complete sets of chromosomes in their bodies formed by physical ways such as temperature and pressure [20], which present advantages of fast growth and good meat quality [21]. Previous studies have shown the difference between triploid and diploid rainbow trout in lipid retention, mobilization [22], and nutrients requirements and utilization [14, 19]. The dietary protein requirements for diploid rainbow trout ranged from 36% to 48% [1], generally 35% to 45% [23], while dietary lipid requirements were 12% [24] or higher than 20% [25–27]. However, few research has been conducted on the nutritional requirements of triploid rainbow trout. The appropriate dietary protein level was 45.8% [14] and 46.76% [28] for 232.8 and 109.03 g triploid rainbow trout in dietary lipid level of 20%, respectively. In a diet with 46% crude protein, for 233 g triploid rainbow trout, the appropriate dietary lipid level would be 23.3% [19] or ≥22.8% [11], respectively. In the above studies, a univariate design was used, and the effects of both dietary protein and lipid (two factors) have not been reported on triploid rainbow trout. Therefore, in this study, three crude lipid levels and two crude protein levels were designed to investigate the effects on growth performance, body composition, feed utilization, lipid metabolism, and antioxidant capacity of triploid rainbow trout. The findings will provide a basis for the development of efficient diets for triploid rainbow trout.

## 2. Material and Methods

### 2.1. Experimental Design and Diets

A 3 × 2 factorial design was conducted with three dietary lipid levels of 4%, 9%, and 14% (L4, L9, and L14) and two dietary protein levels of 44%, 49% (P44, P49), to form six diets as P44/L4, P44/L9, P44/L14, P49/L4, P49/L9, and P49/L14. Fish meal, soybean meal, soy protein concentrate, corn gluten meal, and cottonseed protein concentrate were included in diets as the main protein sources, and fish oil, soybean oil, and soybean phospholipids were used as the main lipid sources. All the diets were balanced by adjusting the content of bentonite and cellulose. All raw materials were finely crushed, sieved through a 60 mesh sieve, and thoroughly mixed in accordance with the feed formula (Table 1). Then, the oil and water were added and the mixture was extruded (temperature of 85 ± 5°C) by a single screw extruder (LX-75 type aquatic feed puffer, Longxiang Food Machinery Factory, Hebei Province, China) to form sinking diets with 3.0 mm diameter. All diets were dried at 50°C until moisture content reached less than 10%, then sealed and stored in a cool and dry place.

### 2.2. Experimental Fish and Feeding Management

Triploid rainbow trout were obtained from the Tiangui Aquaculture Farm in Meishan, Sichuan, China, and transported to the indoor recirculating aquaculture system (RAS) at Binhai Aquaculture Base of Shanghai Ocean University for an 80 day feeding experiment. Before the formal culture trial, the fish were temporarily fed with commercial feed (dietary protein level of 44% and dietary lipid level of 12%) for 2 weeks to adapt to the culture environment. A total of 216 triploid rainbow trout (65.0 ± 0.1 g) were randomly selected and allocated to 18 buckets (1.0 m in diameter and 1.0 m in height, water volume 650 L) with 12 fish per bucket and 3 buckets per treatment. Rainbow trout were hand-fed with diets to apparent satiation twice daily at 10:00 am and 5:00 pm. Culture water was recirculated with a flowing rate of 10 L per minute per bucket and replaced (about 1/3) twice a week. Feces were removed by siphoning in 2 hr after feeding to guarantee the clean water quality. During the culture period, water temperature, dissolved oxygen, pH, ammonia nitrogen, and nitrite were 12–16°C, 6–7 mg/L, 7.0–7.5, ≤0.2 mg/L, and ≤0.1 mg/L, respectively.

### 2.3. Sample Collection

Nine fish were randomly selected and stored at −20°C for whole composition analysis before the feeding trial. After the feeding trial, all fish were deprived of diets for 24 hr, then counted and weighed to calculate WG and FCR. Six fish were randomly selected from each bucket, then anesthetized with MS-222 (30 mg/L), and three fish were stored at −20°C for whole fish composition determination, while another three fish were used to draw blood from caudal vein. The blood was centrifuged at 4,000 r/min for 10 min, and the supernatant was stored at −80°C for biochemical indicators measurements. Immediately after blood collection, the fish were dissected on ice trays, the viscera and liver were weighed for VSI and HSI calculations, and finally the liver and dorsal muscle were frozen at −80°C for subsequent analysis.

### 2.4. Growth Performance and Body Morphometric Indices

WG, FCR, survival rate (SR), HSI, VSI, protein efficiency rate (PER), and protein retention (PR) were calculated as follows:

(1) $$\begin{array}{c} \text{WG}\left(\%\right)=100\times \frac{\left(\text{FBW}-\text{IBW}\right)}{\text{IBW}}, \end{array}$$

(2) $$\begin{array}{c} \text{FCR}=\frac{W_{f}}{\left(\text{FBW}-\text{IBW}\right)}, \end{array}$$

(3) $$\begin{array}{c} \text{SR}\left(\%\right)=100\times \frac{N_{F}}{N_{I}}, \end{array}$$

(4) $$\begin{array}{c} \text{HSI}\left(\%\right)=100\times \left(\frac{W_{L}}{W}\right), \end{array}$$

(5) $$\begin{array}{c} \text{VSI}\left(\%\right)=100\times \left(\frac{W_{V}}{W}\right), \end{array}$$

(6) $$\begin{array}{c} \text{PER}=\frac{\left(\text{FBW}-\text{IBW}\right)}{W_{C}}, \end{array}$$

(7) $$\begin{array}{c} \text{PR}\left(\%\right)=100\times \frac{W_{G}}{W_{C}}. \end{array}$$

FBW, final body weight (g); IBW, initial body weight (g); *W*_*f*_, feed intake (g); NF, final number of fish; *N*_*I*_, initial number of fish; *W*_*L*_, final liver weight (g); *W*, body weight (g); *W*_*V*_, final visceral weight (g); *W*_*C*_, total protein intake (g); and *W*_*G*_, fish protein gain (g).

### 2.5. Proximate Composition of Feed, Whole Fish, Muscle, and Liver

As determined by the AOAC method [30], crude proteins, crude lipids, moisture, and ash were measured by Kjeldahl system method (2300 Auto analyzer; FOSS Tecator, AB, Hoganas, Sweden), chloroform–methanol extraction, oven-drying at 105°C to constant weight and scorching at 550°C in a muffle furnace (SXL-1008 muffle furnace; Shanghai Jinhong Experimental Equipment Co.), respectively.

### 2.6. Fatty Acid Determination

Fatty acids were determined according to the method described by Yang et al. [31]. The lipid was dissolved by adding 2 mL of 14% boron trifluoride–methanol solution. After 25 min of water bath at 100°C, benzene (2 mL) and methanol (2 mL) were added for another water bath (100°C, 25 min). The sample was mixed with distilled water (2 mL) and *n*-hexane (2 mL), and centrifuged at 3,000 rpm/min for 10 min. The supernatant was added with *n*-hexane (0.5 mL) and centrifuged (3,000 rpm/min, 5 min), then the supernatant was collected for the fatty acids analysis with the gas chromatography–mass spectrometry (7980B gas chromatograph-mass spectrometer; Agilent Technologies).

### 2.7. Serum Biochemical Parameters

Serum triglyceride (TG) and total cholesterol (TCHO) levels were determined by the GPO-PAP method and CHOD-PAP method, respectively [32]. High-density lipoprotein cholesterol (HDL-C) and low-density lipoprotein cholesterol (LDL-C) were measured by spectrophotometer. The kits used were purchased from the Nanjing Jiancheng Institute of Biological Engineering.

### 2.8. Liver Biochemical Parameters

Liver tissue was homogenated with nine times of 0.86% saline (ice water bath), then centrifuged at 8,000 r/min for 10 min at 4°C, and the supernatant was collected for the relevant biochemical parameter measurements. Malondialdehyde (MDA) contents, alanine aminotransferase (ALT), and total superoxide dismutase (T-SOD) activities were measured by thiobarbituric acid method, micromethod, and xanthine oxidase method, respectively. Total nitric oxide synthase (T-NOS) and lipase (LPS) activities were determined by colorimetric method. Aspartate aminotransferase (AST), glucose-6-phosphatase (G6P), phosphoenolpyruvate carboxykinase (PEPCK), and catalase (CAT) activities were measured spectrophotometrically. Fatty acid synthase (FAS) and fructose-1,6-bisphosphatase (FBP) activities were measured by the microplate method. The kits used for the experiments were provided by Shanghai Haling Biotechnology Co.

### 2.9. Statistical Analysis

All data were expressed as mean ± standard deviation and analyzed using SPSS 26.0 software. One-way analysis of variance (ANOVA) was used to analyze all data and Tukey's test was chosen for multiple comparisons of significance between groups. Two-way ANOVA was used to analyze the main effects (dietary protein and dietary lipid) and their interactions. *P*-values < 0.05 were considered as statistical significance.

## 3. Results

### 3.1. Growth Performance and Feed Utilization

Dietary lipid significantly affected FBW, WG, FCR, FI, PER, and PR (*P* < 0.05). Fish in P49/L14 group had the highest FI (3.71), WG (374.6%), and the lowest FCR (1.25), while those in P44/L14 group had the highest PER (1.80) and PR (25.06%). The above indicators showed no significant difference between the P49/L14 and P44/L14 groups (*P* > 0.05). Dietary lipid significantly affected the VSI and HSI (*P* < 0.05). The P49/L14 group had the highest VSI (11.27), while the P44/L14 group had the lowest HSI (1.20). The interaction of dietary protein and lipid had no effect (*P* > 0.05) on any of the above indicators.

At the same dietary protein level, WG, PR, PER, and VSI increased, while FCR and HSI decreased as dietary lipid level increased. At the same dietary lipid level, there was no difference (*P* > 0.05) in WG, FCR, PR, PER between P44 and P49 group (Table 2).

### 3.2. Proximate Composition

Dietary lipid significantly affected the moisture content in whole fish, muscle, and crude lipid contents in muscle and liver (*P* < 0.05). As dietary lipid level increased, the moisture content in whole fish and muscle decreased, while the crude lipid contents in muscle and liver increased. The highest muscle and liver lipid contents were found in the P44/L14 and P49/L14 groups, respectively. Dietary protein, dietary lipid, and their interaction had a significant effect on whole fish crude lipid contents (*P* < 0.05), which was increased by the increasing dietary protein and lipid levels. In addition, dietary lipid and the interaction of dietary protein and lipid also significantly affected muscle and liver ash contents (*P* < 0.05). There was no difference observed in the crude protein contents of whole fish, muscle, and liver across all the groups (*P* > 0.05) (Table 3).

### 3.3. Muscle Fatty Acids Composition

Dietary lipid significantly affected *n*-3 PUFAs, *n*-6 PUFAs, PUFAs, and SFAs contents (*P* < 0.05). As dietary lipid level increased, *n*-3 PUFAs, *n*-6 PUFAs, and PUFAs contents increased, while the opposite trend was observed for SFAs. Dietary protein significantly affected muscle *n*-6 PUFAs content (*P* < 0.05). As dietary protein level increased, *n*-6 PUFAs content decreased. In addition, dietary protein, dietary lipid, and their interaction significantly affected MUFAs contents (*P* < 0.05) (Table 4).

### 3.4. Serum Biochemical Indicators

Dietary protein and lipid significantly affected HDL-C levels (*P* < 0.05), which increased as dietary protein and lipid levels increased. Serum TCHO contents were significantly influenced by dietary protein (*P* < 0.05). As dietary protein level increased, TCHO levels increased. Serum LDL-C was significantly affected by dietary lipid (*P* < 0.05), and it increased with increasing the dietary lipid level. Dietary protein, dietary lipid, and their interactions had no effects on TG levels (*P* > 0.05) (Table 5).

### 3.5. Hepatic Metabolic Enzyme Activity

Dietary lipid significantly affected ALT activity, while dietary protein, dietary lipid, and their interaction significantly affected AST activity (*P* < 0.05). The increasing dietary protein level decreased AST activity, while the increasing dietary lipid level decreased both ALT and AST activities.

Dietary protein, dietary lipid, and their interaction significantly affected LPS and FAS activities (*P* < 0.05). LPS and FAS activities increased as dietary protein level increased, and LPS activity also increased as dietary lipid level increased. The increase of dietary lipid level from 4% to 9% significantly increased FAS activity (*P* < 0.05), however, the increase from 9% to 14% did not significantly increase FAS activity (*P* > 0.05).

Dietary protein significantly affected the activity of PEPCK, whereas dietary lipid significantly affected the activities of FBP and G6P (*P* < 0.05). With increasing the dietary protein level, PEPCK activity decreased, while FBP and G6P activities increased with increasing the dietary lipid level (Table 6).

### 3.6. Hepatic Antioxidant Capacity

TNOS activity increased with increasing the dietary protein level, while TSOD, TNOS, and CAT activities were increased by the increasing dietary lipid level. Dietary protein, dietary lipid, and their interaction significantly affected MDA content (*P* < 0.05). In P44 groups, MDA content significantly decreased as dietary lipid level increased, but in P49 groups, MDA content significantly increased as dietary lipid level increased (*P* < 0.05) (Table 7).

## 4. Discussion

### 4.1. Growth Performance and Whole Fish Composition

From a cost-reduction perspective, the use of high-energy feed to reduce dietary protein level is an effective strategy. If diets were deficient in nonprotein energy, protein would be used for energy consumption rather than for growth [33]. In the present study, the P44/L4 group had the lowest WG and highest FCR, which may be due to the insufficient supply of energy and essential fatty acids. In addition, rainbow trout fed diets containing 14% crude lipid showed higher WG, PER, PR, and lower FCR than those fed diets containing 4%, 9% crude lipid, which indicated the protein-sparing effect of dietary lipid [13, 14]. However, the significant increase in whole-body fat contents of rainbow trout (*P* < 0.05) suggested that the sparing effect may be limited under high-fat conditions. It has been reported that the appropriate dietary lipid level for triploid rainbow trout (initial body weight 233 g) cultured in a reservoir cage (water temperature 8–16°C) was 23.3% (crude protein level 46%) [14], and the minimum lipid requirement was 22.8% [11]. The present study was conducted in RAS cultivation (water temperature 12–16°C), and the optimal dietary lipid level of triploid rainbow trout (initial weight of 65 g) was estimated to be 14%, close to the reported lipid requirement (less than 12%) of rainbow trout (initial weight 333.25 g) cultured in RAS (water temperature 14 ± 0.5°C) [34]. The lipid requirement might be affected by fish size and water temperature. Imsland et al. [35] reported that the interaction of water temperature and fish size significantly affected the growth and feed efficiency of Atlantic cod (*Gadus morhua*). Furthermore, the relationship between fish growth and feed was also dependent on water temperature [36]. For example, the appropriate dietary lipid level for optimum growth of rohu (*Labeo rohita*) was 8% and 13%, when the water temperature was 21 and 30°C, respectively [37].

Fish in the rapid growth stage have high protein requirements, and low dietary protein level would reduce the growth rate [38], high dietary protein level could also decrease the growth rate of fish in growth plateau phase [39], and increased feed costs and pollute water quality [16, 17]. In this study, there was no difference in WG, FCR, PR, PER between the P44 and P49 groups at the same dietary lipid level (*P* > 0.05), suggesting that 44% crude protein level might have met the requirement of rainbow trout. The present findings were similar to the reported protein requirement of 45.8% [19] and 46.76% [28] (both at 20% crude lipid level) for triploid rainbow trout.

Fish growth depends on nutrient intakes and utilization [14], and the digestible energy requirement could be met by adjusting FI [40]. Studies on largemouth bass [41] and grass carp (*Ctenopharyngodon idella*) [40] have shown that FI was decreased significantly with increasing the dietary lipid level in diets. In this study, an increase in the level of dietary lipid promoted a significant increase in FI (*P* < 0.05), and the same result was also reported in another study about triploid rainbow trout [14]. Meng et al. [14], suggested that low level of dietary lipid (<12.3%) resulted in reduced feeding activity in rainbow trout. The reason may be related to the different sensitivity of fish species to dietary energy and lipid levels. The differences in stocking density and experimental scale may also affect the requirement [42]. The nutritional status of fish can be reflected by body morphometric indices such as HSI and VSI. In this study, VSI was increased by the increasing dietary lipid level, consistent with the results in whitefish (*Coregonus lavaretus*) [43] and largemouth bass [41]. However, HSI was significantly decreased by the increasing dietary lipid level (*P* < 0.05), and the similar results were also reported in spotted knifejaw (*Oplegnathus punctatus*) [44] and sea bass (*Dicentrarchus labrax*) [45].

### 4.2. Lipid Deposition and Metabolism

Muscle fatty acid composition and fat content are important parameters for assessing the nutritional quality and flavor of fish [46]. Previous studies have shown that rainbow trout flesh with high lipid content may have high quality [11]. In addition, fish flesh with high PUFAs and low SFAs might indicate higher quality for consumers. It has been shown that PUFAs is essential for maintaining cell membrane structure and function [47], while high levels of SFAs may increase the risk of type II diabetes in humans [48]. In the present study, muscle PUFAs content significantly increased and SFAs content decreased with increasing the dietary lipid level (*P* < 0.05), which is consistent with the findings on Senegal sole (*Solea senegalensis*) [49], tilapia (*Oreochromis niloticus*) [50]. Both the PUFAs maxima and SFAs minima were found in the P44/L14 group, suggesting a higher quality of fatty acid composition in the flesh of this group. Compared to DHA, EPA may be preferentially utilized for oxidation [51]. In this study, EPA, DHA, *n*-3 PUFAs, and *n*-6 PUFAs contents increased with increasing the dietary lipid level, but EPA levels were much lower than DHA levels. The possible reason is that the increased dietary lipid level came from the addition of fish oil.

TCHO and TG are important components of blood lipids, which are mainly synthesized in the liver. In Siberian sturgeon (*Acipenser baerii*), the serum TCHO content was not significantly affected by low dietary lipid levels (5.11%, 16.93%), but significantly increased by high dietary lipid level of 20.84% [52]. Similarly, the serum TG contents of hybrid sturgeon (*Acipenser baerii*♀×*A. gueldenstaedtii*♂) were significantly increased by a high dietary lipid level (11.5%) [53]. In the present study, serum TG and TCHO contents were not significantly affected by the increasing dietary lipid level (*P* > 0.05). The possible reason is that dietary lipid level of 14% may be not sufficient to activate endogenous lipid transport in the liver. Studies have shown that lipoprotein levels would respond to feed nutrient levels [54]. With the increase of dietary lipid level from 4% to 16.5%, serum LDL-C was significantly increased in hybrid sturgeon [53]. Ren et al. [52] reported that an increase in dietary lipid level from 5.11% to 16.93% also significantly increased serum HDL-C and LDL-C levels in Siberian sturgeon. In this study, serum HDL-C and LDL-C levels were increased by the increasing dietary lipid level. The increase in serum LDL-C level reflects a decrease in cholesterol deposition in the liver, which may explain the lack of significance in serum TCHO level.

In this study, LPS activity increased with increasing the dietary lipid contents, which reflected the increasing ability of liver to degrade lipid. In general, when exogenous lipid is sufficiently supplied, less lipid would be synthesized in the body with low FAS activity. In largemouth bass [41] and European seabass [55], the FAS activity was significantly decreased by the increasing dietary lipid level. In the present study, liver FAS activity has increased significantly as the dietary lipid level increased from 4% to 9% (*P* < 0.05), but did not further significantly increase when the dietary lipid level increased from 9% to 14% (*P* > 0.05), suggesting that dietary lipid level of 14% might have met the requirement of rainbow trout. In addition, gluconeogenesis is essential for maintaining homeostatic balance of glucose in vertebrates [56]. The present result was consistent with the findings on largemouth bass [41], where an increase in the dietary lipid level was accompanied by an increase in FBP and PEPCK activities, reflecting an improved gluconeogenesis process.

### 4.3. Antioxidant Capacity and Transaminase Activity of the Liver

SOD, NOS, and CAT activities are often used to assess the effect of dietary lipid on the antioxidant capacity of tissues [41, 57]. In this study, T-SOD, T-NOS, and CAT activities increased as dietary lipid level increased, suggesting that the increased dietary lipid level exacerbated lipid oxidation, thus, antioxidant enzyme activity was promoted. Also, as the increase of dietary lipid level came from the increase of fish oil, the high level of HUFA from fish oil may also exacerbate the production of ROS [41]. In the present study, T-NOS activity increased as dietary protein level increased, while T-SOD activity did not significantly change, which reflected that high protein level may exacerbate lipid deposition in the liver. As one important product of fatty acid peroxidation in the liver, MDA content can be used to reflect the oxidation degree in tissues [41]. In this study, MDA content significantly decreased as dietary lipid level increased in P44 diets, while in P49 diets, MDA significantly increased with increasing the dietary lipid level (*P* < 0.05), indicating that high lipid level exacerbated the lipid oxidation of rainbow trout fed a high protein diet, but not the fish fed a low protein diet.

As the main site of protein metabolism in fish, AST and ALT activities in the liver can reflect the status of protein metabolism. In general, the increase of digestible protein in the diet would increase the AST and ALT activities in the liver [58, 59]. However, in white sea bream (*Diplodus sargus*), AST and ALT activities did not further increase with increasing the dietary protein level when dietary protein level has met the requirement [60]. In this study, AST activity decreased with increasing the dietary protein level. Similar to the findings on rohu [61] and brown trout [62], the AST and ALT activities were also decreased with increasing the dietary lipid level in this study, reflecting the protein-sparing effects of dietary lipid. However, FAS and LPS activities also increased with increasing the dietary protein level, which indicated that high protein diets may burden the lipid deposition in the liver.

## 5. Conclusion

The present results showed that increasing the dietary lipid level from 4% to 14% significantly improved the growth performance and feed utilization of triploid rainbow trout with an initial weight of 65.0 g, but the increase of dietary protein level from 44% to 49% did not significantly affect the growth performance, while increased whole fish lipid content and reduced PR. The P44/L14 group had the highest PER, PR, whole fish protein, *n*-3 PUFAs, *n*-6 PUFAs, and PUFAs. Therefore, based on growth performance and feed utilization, dietary protein level of 44% and dietary lipid level of 14% (measured value, 43.71%, 13.62%) were suggested for young triploid rainbow trout.

## Figures and Tables

**Table 1:** Diet formulation and proximate composition (air dry basis, g/kg).

Ingredients	P44/L4	P44/L9	P44/L14	P49/L4	P49/L9	P49/L14
Fish meal	200.0	200.0	200.0	240.0	240.0	240.0
Soybean meal	120.0	120.0	120.0	133.0	133.0	133.0
Soy protein concentrate	80.0	84.0	88.0	100.0	103.5	107.0
Wheat flour	287.0	245.4	203.8	187.0	145.4	103.8
Corn gluten meal	50.0	52.0	54.0	60.0	63.0	66.0
Cottonseed protein concentrate	80.0	84.0	88.0	100.0	103.5	107.0
Meat meal	50.0	50.0	50.0	50.0	50.0	50.0
Beer yeast	50.0	50.0	50.0	50.0	50.0	50.0
Fish oil	1.0	18.2	35.4	0.0	17.2	34.4
Soybean oil	1.0	18.2	35.4	0.0	17.2	34.4
Soybean phospholipids	1.0	18.2	35.4	0.0	17.2	34.4
Vitamin premix^a^	10.0	10.0	10.0	10.0	10.0	10.0
Mineral complex^b^	30.0	30.0	30.0	30.0	30.0	30.0
Bentonite	20.0	10.0	0.0	20.0	10.0	0.0
Cellulose	20.0	10.0	0.0	20.0	10.0	0.0
Total	1,000.0	1,000.0	1,000.0	1,000.0	1,000.0	1,000.0
Proximate composition						
Crude protein	444.1	443.5	437.1	492.5	491.3	487.6
Crude lipid	52.4	83.7	136.2	45.5	90.9	142.4
Ash	140.1	132.1	123.2	146.6	137.7	128.1
Moisture	84.9	83.2	81.0	85.9	82.9	81.9
Gross energy (calculated value; MJ/kg)^c^	17.3	18.2	19.5	17.4	18.6	19.9

*Note*: ^a^Vitamin premix (IU/kg diet): VA, 10,000; VD3, 3,000; and VE, 150. Vitamin premix (mg/kg diet): VK_3_, 12.17; VB_1_, 20; VB_2_, 20; VB_3_, 100; VB_6_, 22; VB_12_, 0.15; VC, 300; biotin, 0.6; inositol, 400; and folic acid, 8. ^b^Mineral premix (mg/kg diet): I, 1.5; Mn, 11.45; Co, 0.6; Cu, 3; Zn, 89; Se, 0.24; Mg, 180; and Fe, 63. ^c^Gross energy = 23.6 × Crude protein+39.5 × Crude lipid+17.2 × Carbohydrate [29].

**Table 2:** Dietary effects of lipid and protein levels on growth performance and feed utilization of triploid *Oncorhynchus mykiss*.

Diet	IBW (g)	FBW (g)	WG (%)	FI (g/fish/day)	SR (%)	VSI (%)	HSI (%)	FCR	PER	PR (%)
P44/L4	64.9 ± 0.1	261.0 ± 8.8^c^	294.9 ± 10.2^d^	3.60 ± 0.01^c^	100	9.37 ± 0.3^b^	1.35 ± 0.08^ab^	1.54 ± 0.07^a^	1.47 ± 0.07^c^	20.99 ± 0.77^bc^
P44/L9	65.1 ± 0.1	274.3 ± 5.6^bc^	322.0 ± 8.6^bcd^	3.66 ± 0.01^b^	100	10.48 ± 0.7^ab^	1.29 ± 0.12^abc^	1.46 ± 0.04^ab^	1.55 ± 0.04^bc^	21.98 ± 0.01^abc^
P44/L14	65.0 ± 0.1	304.0 ± 16.5^ab^	367.7 ± 25.5^ab^	3.69 ± 0.03^ab^	100	10.74 ± 0.4^ab^	1.20 ± 0.06^cd^	1.28 ± 0.09^c^	1.80 ± 0.12^a^	25.06 ± 1.27^a^
P49/L4	65.0 ± 0.2	271.4 ± 5.5^c^	309.1 ± 15.7^cd^	3.61 ± 0.01^c^	100	9.48 ± 0.3^b^	1.54 ± 0.13^ab^	1.50 ± 0.08^ab^	1.36 ± 0.07^c^	19.19 ± 0.98^c^
P49/L9	65.1 ± 0.2	291.3 ± 4.8^abc^	348.1 ± 7.4^abc^	3.67 ± 0.02^ab^	100	10.02 ± 0.6^ab^	1.28 ± 0.12^bcd^	1.33 ± 0.03^bc^	1.53 ± 0.03^bc^	21.12 ± 1.15^bc^
P49/L14	65.1 ± 0.1	308.5 ± 11.5^a^	374.6 ± 17.7^a^	3.71 ± 0.01^a^	100	11.27 ± 1.0^a^	1.25 ± 0.06^d^	1.25 ± 0.06^c^	1.70 ± 0.12^ab^	24.56 ± 1.10^ab^
*Means of main effects*										
Dietary protein (%)										
44	65.0 ± 0.1	279.8 ± 21.4	332.4 ± 34.7	3.65 ± 0.04	–	10.20 ± 0.76	1.28 ± 0.10	1.42 ± 0.13	1.60 ± 0.17	22.67 ± 2.01
49	65.1 ± 0.1	290.5 ± 16.3	340.1 ± 30.1	3.66 ± 0.04	–	10.26 ± 0.98	1.32 ± 0.17	1.37 ± 0.12	1.53 ± 0.17	21.62 ± 2.84
Dietary lipid (%)										
4	65.1 ± 0.1	265.2 ± 8.9^c^	303.5 ± 14.5^c^	3.61 ± 0.01^c^	–	9.42 ± 0.27^c^	1.43 ± 0.14^a^	1.52 ± 0.07^a^	1.41 ± 0.09^c^	20.09 ± 1.26^b^
9	65.0 ± 0.0	282.8 ± 10.4^b^	335.0 ± 16.0^b^	3.67 ± 0.01^b^	–	10.25 ± 0.63^ab^	1.28 ± 0.11^ab^	1.39 ± 0.08^b^	1.54 ± 0.03^b^	21.55 ± 0.82^b^
14	65.0 ± 0.2	305.8 ± 13.3^a^	370.4 ± 20.4^a^	3.70 ± 0.02^a^	–	11.01 ± 0.71^a^	1.21 ± 0.07^b^	1.27 ± 0.07^c^	1.75 ± 0.12^a^	24.81 ± 1.01^a^
Two-way ANOVA										
Dietary protein	0.666	0.062	0.078	0.139	–	0.836	0.184	0.062	0.079	0.110
Dietary lipid	0.528	0.000	0.000	0.000	–	0.002	0.009	0.000	0.000	0.001
Dietary protein × lipid	0.571	0.600	0.611	0.899	–	0.370	0.264	0.337	0.551	0.642

*Note*: FBW, final body weight; FCR, feed conversion ratio; FI, feed intake; HSI, hepatosomatic index; IBW, initial body weight; PER, protein efficiency rate; PR, protein retention rate; SR, survival rate; VSI, viscerosomatic index; WG, weight gain. Within multiple comparisons or main effects analysis, data with different small letter superscripts indicate significant differences (*P* < 0.05), while data for two-factor analysis of variance are less than 0.05, indicating significant differences (*P* < 0.05). Data are expressed as mean ± standard deviation.

**Table 3:** Dietary effects of lipid and protein levels on proximate composition in whole fish, muscle, and liver of triploid *Oncorhynchus mykiss* (wet weight, g/kg).

Diet	Whole fish (g/kg)				Muscle (g/kg)				Liver (g/kg)			
	Moisture	Crude protein	Crude lipid	Ash	Moisture	Crude protein	Crude lipid	Ash	Moisture	Crude protein	Crude lipid	Ash
P44/L4	709.1 ± 27.4^a^	152.1 ± 2.36	60.8 ± 2.3^c^	20.5 ± 0.1	769.5 ± 4.0^a^	158.6 ± 1.1	34.6 ± 2.4^ab^	8.0 ± 0.4^bc^	672.4 ± 8.8^a^	177.1 ± 4.4	65.0 ± 5.8^c^	15.4 ± 0.3^ab^
P44/L9	677.9 ± 12.2^ab^	144.3 ± 1.77	80.4 ± 2.2^b^	22.0 ± 1.1	764.6 ± 4.8^abc^	159.8 ± 5.0	39.2 ± 2.2^ab^	6.7 ± 0.2^cd^	676.3 ± 7.3^a^	180.8 ± 4.3	76.0 ± 3.6^abc^	14.2 ± 0.8^b^
P44/L14	673.8 ± 6.5^ab^	150.4 ± 9.38	98.2 ± 1.2^a^	19.6 ± 0.8	752.4 ± 10.3^bc^	165.8 ± 8.7	42.3 ± 1.2^a^	9.3 ± 0.4^b^	630.8 ± 2.2^b^	189.1 ± 3.18	76.8 ± 0.9^abc^	14.4 ± 0.7^b^
P49/L4	697.1 ± 14.3^ab^	145.4 ± 3.85	78.5 ± 3.6^b^	21.1 ± 0.2	767.5 ± 4.7^ab^	163.9 ± 6.8	33.5 ± 2.6^b^	11.1 ± 0.2^a^	675.4 ± 14.6^a^	180.0 ± 8.0	68.0 ± 4.0^bc^	15.3 ± 0.2^ab^
P49/L9	687.2 ± 2.15^ab^	145.8 ± 2.83	83.4 ± 4.2^b^	19.7 ± 0.7	757.5 ± 3.8^abc^	166.2 ± 2.7	37.3 ± 3.2^ab^	7.4 ± 0.5^cd^	677.4 ± 4.0^a^	183.3 ± 3.8	79.2 ± 3.5^ab^	16.7 ± 0.3^a^
P49/L14	659.4 ± 4.9^b^	147.1 ± 2.03	108.0 ± 0.6^a^	21.0 ± 1.8	750.2 ± 0.6^c^	166.6 ± 4.3	41.3 ± 3.5^ab^	6.3 ± 0.2^d^	673.2 ± 10.3^a^	177.3 ± 7.1	85.2 ± 1.2^a^	15.8 ± 0.1^ab^
*Means of main effects*												
Dietary protein (%)												
44	687.0 ± 22.7	147.4 ± 6.5	79.8 ± 17.1^b^	20.5 ± 1.3	763.2 ± 9.3	161.0 ± 5.3	38.1 ± 3.9	8.1 ± 1.1	661.6 ± 21.9	180.0 ± 7.2	72.6 ± 6.7^b^	15.3 ± 1.1
49	681.2 ± 18.6	146.2 ± 2.7	92.8 ± 14.0^a^	20.6 ± 1.1	760.5 ± 9.4	165.3 ± 4.6	36.9 ± 4.1	8.3 ± 2.3	675.4 ± 9.4	180.2 ± 6.2	76.1 ± 8.5^a^	15.0 ± 0.8
Dietary lipid (%)												
4	703.1 ± 20.6^a^	148.8 ± 4.6	67.5 ± 11.8^c^	20.7 ± 0.4	769.9 ± 4.2^a^	161.3 ± 5.2	34.1 ± 2.3^b^	9.6 ± 1.8^a^	673.9 ± 10.9^a^	178.5 ± 6.0	66.8 ± 4.4^b^	14.7 ± 0.9^b^
9	682.6 ± 9.4^ab^	145.0 ± 2.1	81.9 ± 4.4^b^	20.9 ± 1.5	760.3 ± 5.3^b^	163.0 ± 4.9	38.1 ± 2.7^ab^	7.4 ± 0.9^b^	677.0 ± 4.6^a^	182.0 ± 3.8	77.6 ± 3.5^a^	14.8 ± 0.7^b^
14	666.6 ± 9.4^b^	146.0 ± 6.2	103.1 ± 5.7^a^	20.2 ± 1.3	751.3 ± 6.1^b^	166.2 ± 5.6	41.8 ± 2.2^a^	7.8 ± 1.8^b^	656.3 ± 24.4^b^	179.7 ± 9.3	81.0 ± 4.9^a^	16.2 ± 0.6^a^
Two-way ANOVA												
Dietary protein	0.402	0.706	0.000	0.876	0.336	0.185	0.368	0.260	0.009	0.954	0.049	0.173
Dietary lipid	0.003	0.466	0.000	0.664	0.001	0.394	0.004	0.000	0.004	0.709	0.001	0.004
Dietary protein × lipid	0.305	0.277	0.000	0.063	0.497	0.734	0.963	0.000	0.010	0.600	0.520	0.018

*Note*: Within multiple comparisons or main effects analysis, data with different small letter superscripts indicate significant differences (*P* < 0.05), while data for two-factor analysis of variance are less than 0.05, indicating significant differences (*P* < 0.05). Data are expressed as mean ± standard deviation.

**Table 4:** Dietary effects of lipid and protein levels on fatty acid composition in muscle of triploid *Oncorhynchus mykiss* (percentage of total fatty acids, %).

Parameters	P44			P49			Two-way ANOVA		
	L4	L9	L14	L4	L9	L14	Dietary protein	Dietary lipid	Dietary protein × lipid
C14:0	1.68 ± 0.03^a^	1.46 ± 0.07^b^	1.30 ± 0.13^b^	1.84 ± 0.06^a^	1.48 ± 0.09^b^	1.47 ± 0.02^b^	0.005	0.000	0.159
C16:0	19.6 ± 0.18^a^	16.58 ± 0.71^b^	15.47 ± 0.30^b^	19.45 ± 0.5^a^	16.72 ± 0.83^b^	16.71 ± 0.58^b^	0.181	0.000	0.175
C17:0	0.20 ± 0.00^b^	0.29 ± 0.02^a^	0.28 ± 0.04^a^	0.19 ± 0.01^b^	0.28 ± 0.01^a^	0.29 ± 0.02^a^	0.858	0.000	0.455
C18:0	4.38 ± 0.21^ab^	4.31 ± 0.23^ab^	3.94 ± 0.11^b^	4.83 ± 0.32^a^	4.47 ± 0.32^ab^	4.31 ± 0.18^ab^	0.020	0.029	0.603
SFAs	25.63 ± 0.49^a^	22.64 ± 1.00^b^	20.98 ± 0.49^b^	26.18 ± 0.87^a^	22.96 ± 1.18^b^	22.79 ± 0.58^b^	0.053	0.000	0.342
C16:*n*-9	1.00 ± 0.08^a^	0.61 ± 0.05^c^	0.54 ± 0.05^c^	0.85 ± 0.08^ab^	0.67 ± 0.01^bc^	0.69 ± 0.05^bc^	0.382	0.000	0.232
C16:*n*-7	8.66 ± 0.55^a^	4.96 ± 0.16^b^	3.59 ± 0.07^b^	8.46 ± 0.86^a^	4.92 ± 0.03^b^	5.34 ± 0.34^b^	0.116	0.000	0.047
C18:*n*-9	39.68 ± 1.10^a^	33.47 ± 1.89^b^	29.39 ± 0.67^b^	40.98 ± 0.60^a^	32.20 ± 2.84^b^	32.18 ± 2.35^b^	0.323	0.000	0.232
C20:*n*-9	1.29 ± 0.04^b^	1.16 ± 0.09^bc^	1.01 ± 0.04^c^	1.63 ± 0.16^a^	1.16 ± 0.11^bc^	1.17 ± 0.07^bc^	0.954	0.709	0.600
MUFAs	49.58 ± 0.04^b^	41.35 ± 0.61^c^	34.44 ± 0.42^d^	51.61 ± 0.37^a^	40.62 ± 0.84^c^	40.77 ± 0.40^c^	0.000	0.000	0.000
C18:3*n*-3	0.81 ± 0.05^c^	1.74 ± 0.05^b^	2.44 ± 0.01^a^	0.75 ± 0.05^c^	1.72 ± 0.00^b^	1.78 ± 0.02^b^	0.000	0.000	0.000
C20:5*n*-3	1.08 ± 0.08^c^	1.53 ± 0.06^a^	1.53 ± 0.02^a^	1.22 ± 0.03^bc^	1.35 ± 0.06^ab^	1.25 ± 0.01^bc^	0.013	0.000	0.003
C22:6*n*-3	6.24 ± 0.43	6.83 ± 0.45	7.57 ± 0.56	6.69 ± 0.55	7.11 ± 0.08	7.04 ± 0.55	0.642	0.034	0.213
*n*-3 PUFAs	8.17 ± 0.54^b^	10.18 ± 0.89^ab^	11.52 ± 0.54^a^	8.69 ± 0.59^b^	10.40 ± 0.23^ab^	10.40 ± 1.12^ab^	0.800	0.000	0.193
C18:2*n*-6	13.68 ± 0.29^c^	22.59 ± 0.02^b^	29.36 ± 0.40^a^	11.18 ± 0.64^d^	22.19 ± 0.64^b^	23.07 ± 0.11^b^	0.000	0.000	0.000
C18:3*n*-6	0.34 ± 0.01^b^	0.33 ± 0.02^bc^	0.41 ± 0.04^a^	0.27 ± 0.03^c^	0.34 ± 0.02^b^	0.37 ± 0.02^ab^	0.015	0.000	0.053
C20:2*n*-6	0.77 ± 0.05^c^	1.14 ± 0.07^b^	1.38 ± 0.06^a^	0.64 ± 0.06^c^	1.18 ± 0.05^b^	1.19 ± 0.05^b^	0.012	0.000	0.026
C20:3*n*-6	0.58 ± 0.03^bc^	0.63 ± 0.03^b^	0.77 ± 0.00^a^	0.49 ± 0.05^c^	0.69 ± 0.01^ab^	0.70 ± 0.06^ab^	0.113	0.000	0.022
C20:4*n*-6	0.91 ± 0.04^c^	0.99 ± 0.05^bc^	1.19 ± 0.05^a^	0.89 ± 0.04^c^	0.99 ± 0.02^bc^	1.08 ± 0.03^ab^	0.039	0.000	0.107
*n*-6 PUFAs	16.19 ± 0.13^c^	25.68 ± 0.02^b^	33.09 ± 0.45^a^	13.53 ± 0.67^d^	25.38 ± 0.59^b^	26.42 ± 0.07^b^	0.025	0.000	0.227
PUFAs	24.57 ± 0.42^c^	35.44 ± 0.78^b^	44.61 ± 0.11^a^	21.62 ± 0.42^d^	35.77 ± 0.37^b^	36.18 ± 0.13^b^	0.060	0.000	0.194
DHA/EPA	5.55 ± 0.31	4.85 ± 0.34	4.97 ± 0.44	5.34 ± 0.33	5.24 ± 0.46	5.10 ± 0.47	0.598	0.173	0.440

*Note*: DHA, C22:6*n*-3; EPA, C20:5*n*-3; MUFAs, monounsaturated fatty acids; *n*-3 PUFAs, *n*-3 polyunsaturated fatty acids; *n*-6 PUFA, *n*-6 polyunsaturated fatty acids; PUFAs, polyunsaturated fatty acids; SFAs, saturated fatty acids. Within multiple comparisons or main effects analysis, data with different small letter superscripts indicate significant differences (*P* < 0.05), while data for two-factor analysis of variance are less than 0.05, indicating significant differences (*P* < 0.05). Data are expressed as mean ± standard deviation.

**Table 5:** Dietary effects of lipid and protein levels on serum biochemical indicators of triploid *Oncorhynchus mykiss*.

Diet	TG (mmol/L)	TCHO (mmol/L)	HDL-C (mmol/L)	LDL-C (mmol/L)
P44/L4	1.12 ± 0.11	1.82 ± 0.14^b^	3.30 ± 0.42^d^	1.69 ± 0.13
P44/L9	0.91 ± 0.09	1.86 ± 0.03^b^	4.35 ± 0.23^c^	1.91 ± 0.12
P44/L14	1.16 ± 0.01	1.84 ± 0.04^b^	5.19 ± 0.11^abc^	2.07 ± 0.08
P49/L4	0.99 ± 0.13	2.00 ± 0.06^ab^	4.60 ± 0.32^bc^	1.77 ± 0.14
P49/L9	0.96 ± 0.11	1.90 ± 0.03^ab^	5.63 ± 0.07^a^	1.78 ± 0.08
P49/L14	1.04 ± 0.02	2.12 ± 0.06^a^	5.33 ± 0.17^ab^	1.93 ± 0.12
*Means of main effects*				
Dietary protein (%)				
44	1.06 ± 0.14	1.84 ± 0.07^b^	4.42 ± 0.76^b^	1.89 ± 0.18
49	1.00 ± 0.09	2.01 ± 0.11^a^	5.08 ± 0.66^a^	1.82 ± 0.12
Dietary lipid (%)				
4	1.04 ± 0.13	1.91 ± 0.14	4.01 ± 0.65^b^	1.73 ± 0.12^b^
9	0.93 ± 0.09	1.88 ± 0.03	5.13 ± 0.64^a^	1.86 ± 0.12^ab^
14	1.10 ± 0.07	1.95 ± 0.16	5.11 ± 0.36^a^	1.99 ± 0.12^a^
Two-way ANOVA				
Dietary protein	0.281	0.003	0.010	0.361
Dietary lipid	0.111	0.140	0.002	0.037
Dietary protein × lipid	0.369	0.087	0.547	0.359

*Note*: HDL-C, high-density lipoprotein cholesterol; LDL-C, low-density lipoprotein cholesterol; TCHO, total cholesterol; TG, triglyceride. Within multiple comparisons or main effects analysis, data with different small letter superscripts indicate significant differences (*P* < 0.05), while data for two-factor analysis of variance are less than 0.05, indicating significant differences (*P* < 0.05). Data are expressed as mean ± standard deviation.

**Table 6:** Dietary effects of lipid and protein levels on liver enzymes related to nutritional metabolism of triploid *Oncorhynchus mykiss* liver.

Diet	Protein metabolism		Lipid metabolism		Gluconeogenesis		
	AST (U/g mass)	ALT (U/g mass)	LPS (U/g prot)	FAS (U/mg prot)	G6P (U/mg prot)	FBP (U/mg prot)	PEPCK (U/mg prot)
P44/L4	54.60 ± 0.74^a^	36.12 ± 0.21^a^	136.80 ± 6.09^b^	1.61 ± 0.06^d^	8.78 ± 0.85	1.88 ± 0.09^bc^	62.45 ± 3.07^a^
P44/L9	47.58 ± 2.03^bc^	32.15 ± 2.16^ab^	162.42 ± 9.55^b^	1.77 ± 0.02^c^	9.84 ± 0.11	2.06 ± 0.01^ab^	64.13 ± 1.03^a^
P44/L14	48.87 ± 2.07^ab^	30.77 ± 1.19^b^	164.01 ± 11.07^b^	1.81 ± 0.01^c^	10.56 ± 0.64	2.18 ± 0.02^a^	64.38 ± 0.39^a^
P49/L4	44.75 ± 0.39^bc^	35.66 ± 0.21^ab^	148.14 ± 11.38^b^	2.07 ± 0.02^b^	9.53 ± 0.56	1.85 ± 0.04^c^	54.12 ± 1.20^b^
P49/L9	44.84 ± 2.07^bc^	33.37 ± 2.60^ab^	163.20 ± 6.80^b^	2.43 ± 0.06^a^	10.74 ± 0.06	1.99 ± 0.07^abc^	54.26 ± 0.59^b^
P49/L14	42.10 ± 2.07^c^	33.29 ± 0.54^ab^	206.87 ± 10.13^a^	2.47 ± 0.03^a^	11.04 ± 0.99	2.19 ± 0.01^a^	55.51 ± 0.86^b^
*Means of main effects*							
Dietary protein (%)							
44	50.54 ± 3.68^a^	32.69 ± 2.67	154.41 ± 15.42^b^	1.73 ± 0.10^b^	9.59 ± 0.98	2.04 ± 0.14	63.65 ± 1.73^a^
49	43.89 ± 1.92^b^	34.11 ± 1.69	169.22 ± 27.83^a^	2.32 ± 0.20^a^	10.44 ± 0.88	2.01 ± 0.16	54.63 ± 0.99^b^
Dietary lipid (%)							
4	50.66 ± 5.43^a^	35.89 ± 0.32^a^	142.47 ± 10.26^c^	1.84 ± 0.27^b^	9.08 ± 0.78^b^	1.87 ± 0.06^c^	58.28 ± 5.17
9	46.48 ± 2.33^b^	32.76 ± 2.07^b^	162.73 ± 7.57^b^	2.10 ± 0.39^a^	10.29 ± 0.53^ab^	2.03 ± 0.06^b^	59.19 ± 5.74
14	45.48 ± 4.26^b^	31.78 ± 1.64^b^	181.16 ± 25.26^a^	2.14 ± 0.38^a^	10.80 ± 0.74^a^	2.18 ± 0.02^a^	59.94 ± 5.15
Two-way ANOVA							
Dietary protein	0.000	0.219	0.004	0.000	0.101	0.323	0.000
Dietary lipid	0.012	0.013	0.000	0.000	0.021	0.000	0.349
Dietary protein × lipid	0.032	0.366	0.016	0.016	0.607	0.526	0.766

*Note*: ALT, alanine aminotransferase; AST, aspartate aminotransferase; FAS, fatty acid synthase; FBP, fructose-1,6-bisphosphatase; G6P, glucose-6-phosphatase; LPS, lipase; PEPCK, phosphoenolpyruvate carboxykinase. Within multiple comparisons or main effects analysis, data with different small letter superscripts indicate significant differences (*P* < 0.05), while data for two-factor analysis of variance are less than 0.05, indicating significant differences (*P* < 0.05). Data are expressed as mean ± standard deviation.

**Table 7:** Dietary effects of lipid and protein levels on antioxidant capacity in the liver of triploid *Oncorhynchus mykiss*.

Diet	TSOD (U/mg prot)	CAT (U/mg prot)	TNOS (U/mg prot)	MDA (nmol/mg prot)
P44L4	72.24 ± 9.25^c^	13.20 ± 2.04^b^	623.57 ± 48.72^c^	0.88 ± 0.05^a^
P44L9	84.90 ± 4.03^abc^	18.56 ± 2.73^ab^	647.48 ± 22.42^bc^	0.61 ± 0.01^b^
P44L14	93.77 ± 4.48^ab^	20.73 ± 3.30^a^	756.44 ± 7.07^b^	0.47 ± 0.04^c^
P49L4	78.44 ± 9.88^bc^	12.95 ± 1.13^b^	739.82 ± 49.04^bc^	0.62 ± 0.01^b^
P49L9	88.36 ± 4.97^abc^	18.18 ± 2.84^ab^	748.37 ± 42.41^bc^	0.76 ± 0.03^a^
P49L14	103.38 ± 9.06^a^	19.78 ± 2.55^ab^	899.70 ± 43.68^a^	0.78 ± 0.01^a^
*Means of main effects*				
Dietary protein (%)				
44	83.64 ± 10.88	17.50 ± 4.11	679.37 ± 70.25^b^	0.68 ± 0.19^b^
49	90.06 ± 13.01	16.97 ± 3.68	810.78 ± 90.92^a^	0.72 ± 0.08^a^
Dietary lipid (%)				
4	75.34 ± 9.21^b^	13.08 ± 1.48^b^	670.07 ± 76.43^b^	0.78 ± 0.15^a^
9	86.63 ± 4.46^b^	18.37 ± 2.50^a^	697.92 ± 64.50^b^	0.68 ± 0.09^b^
14	98.58 ± 8.28^a^	20.26 ± 2.68^a^	828.07 ± 83.31^a^	0.62 ± 0.08^b^
Two-way ANOVA				
Dietary protein	0.089	0.666	0.000	0.002
Dietary lipid	0.001	0.001	0.000	0.006
Dietary protein × lipid	0.773	0.968	0.688	0.000

*Note*: CAT, catalase; MDA, malondialdehyde; TNOS, total nitric oxide; TSOD, total superoxide dismutase. Within multiple comparisons or main effects analysis, data with different small letter superscripts indicate significant differences (*P* < 0.05), while data for two-factor analysis of variance are less than 0.05, indicating significant differences (*P* < 0.05). Data are expressed as mean ± standard deviation.

## Data Availability

All data generated or analyzed during this study are included in this article.

## References

[1] NRC (2011). *Nutrient Requirements of Fish and Shrimp*.

[2] Habte-Tsion H.-M., Liu B., Ge X. (2013). Effects of dietary protein level on growth performance, muscle composition, blood composition, and digestive enzyme activity of Wuchang bream (*Megalobrama amblycephala*) fry. *The Israeli Journal of Aquaculture—Bamidgeh*.

[3] Li X.-F., Liu W.-B., Jiang Y.-Y., Zhu H., Ge X.-P. (2010). Effects of dietary protein and lipid levels in practical diets on growth performance and body composition of blunt snout bream (*Megalobrama amblycephala*) fingerlings. *Aquaculture*.

[4] Güroy D., Şahin I., Güroy B., Altin A., Merrifield D. L. (2012). Effect of dietary protein level on growth performance and nitrogen excretion of the yellow tail cichlid, *Pseudotropheus acei*. *The Israeli Journal of Aquaculture—Bamidgeh*.

[5] Watanabe T. (1982). Lipid nutrition in fish. *Comparative Biochemistry and Physiology Part B: Comparative Biochemistry*.

[6] Sargent J., McEvoy L., Estevez A. (1999). Lipid nutrition of marine fish during early development: current status and future directions. *Aquaculture*.

[7] Jauncey K. (1982). The effects of varying dietary protein level on the growth, food conversion, protein utilization and body composition of juvenile tilapias (*Sarotherodon mossambicus*). *Aquaculture*.

[8] Sun P., Jin M., Ding L. (2018). Dietary lipid levels could improve growth and intestinal microbiota of juvenile swimming crab, *Portunus trituberculatus*. *Aquaculture*.

[9] Regost C., Arzel J., Cardinal M., Laroche M., Kaushik S. J. (2001). Fat deposition and flesh quality in seawater reared, triploid brown trout (*Salmo trutta*) as affected by dietary fat levels and starvation. *Aquaculture*.

[10] Nickell D. C., Bromage N. R. (1998). The effect of dietary lipid level on variation of flesh pigmentation in rainbow trout (*Oncorhynchus mykiss*). *Aquaculture*.

[11] Meng Y., Liu X., Guan L. (2023). Does dietary lipid level affect the quality of triploid rainbow trout and how should it be assessed?. *Foods*.

[12] Dabrowski K., Guderley H. (2002). Intermediary metabolism. *Fish Nutrition*.

[13] Karalazos V., Bendiksen E., Bell J. G. (2011). Interactive effects of dietary protein/lipid level and oil source on growth, feed utilisation and nutrient and fatty acid digestibility of Atlantic salmon. *Aquaculture*.

[14] Meng Y., Qian K., Ma R. (2019). Effects of dietary lipid levels on sub-adult triploid rainbow trout (*Oncorhynchus mykiss*): 1. Growth performance, digestive ability, health status and expression of growth-related genes. *Aquaculture*.

[15] Huang D., Wu Y., Lin Y. (2017). Dietary protein and lipid requirements for juvenile largemouth bass, *Micropterus salmoides*. *Journal of the World Aquaculture Society*.

[16] Wang J. T., Han T., Li X. Y. (2017). Effects of dietary protein and lipid levels with different protein-to-energy ratios on growth performance, feed utilization and body composition of juvenile red-spotted grouper, *Epinephelus akaara*. *Aquaculture Nutrition*.

[17] Ahmed I., Ahmad I. (2020). Effect of dietary protein levels on growth performance, hematological profile and biochemical composition of fingerlings rainbow trout, *Oncorhynchus mykiss* reared in Indian Himalayan region. *Aquaculture Reports*.

[18] Food and Agriculture Organization of the United Nations (FAO) (2020). *The State of World Fisheries and Aquaculture 2020. Sustainability in Action*.

[19] Ma R., Liu X., Meng Y. (2019). Protein nutrition on sub-adult triploid rainbow trout (1): dietary requirement and effect on anti-oxidative capacity, protein digestion and absorption. *Aquaculture*.

[20] Salimian S., Keyvanshokooh S., Salati A. P., Pasha-Zanoosi H., Babaheydari S. B. (2016). Effects of triploidy induction on physiological and immunological characteristics of rainbow trout (*Oncorhynchus mykiss*) at early developmental stages (fertilized eggs, eyed eggs and fry). *Animal Reproduction Science*.

[21] Huang T., Sun H., Wang Y., Xu G., Wang X., Han Y. (2018). Effect of follicle cell autophagy on gonadal development of triploid female rainbow trout (*Oncorhynchus mykiss*). *Fish Physiology and Biochemistry*.

[22] Manor M. L., Cleveland B. M., Weber G. M., Kenney P. B. (2015). Effects of sexual maturation and feeding level on fatty acid metabolism gene expression in muscle, liver, and visceral adipose tissue of diploid and triploid rainbow trout, *Oncorhynchus mykiss*. *Comparative Biochemistry and Physiology Part B: Biochemistry and Molecular Biology*.

[23] Eliason E. J., Higgs D. A., Farrell A. P. (2007). Effect of isoenergetic diets with different protein and lipid content on the growth performance and heat increment of rainbow trout. *Aquaculture*.

[24] National Research Council (NRC) (1981). *Nutrient Requirements of Coldwater Fishes*.

[25] Hecht T., McEwan A. G. (1992). Change in gross protein and lipid requirements of rainbow trout at elevated temperatures. *Aquaculture*.

[26] Luzzana U., Serrini G., Moretti V. M., Gianesini C., Valfrè F. (1994). Effect of expanded feed with high fish oil content on growth and fatty acid composition of rainbow trout. *Aquaculture International*.

[27] Gélineau A., Corraze G., Boujard T., Larroquet L., Kaushik S. (2001). Relation between dietary lipid level and voluntary feed intake, growth, nutrient gain, lipid deposition and hepatic lipogenesis in rainbow trout. *Reproduction Nutrition Development*.

[28] Liu G., Wang L., Yu H. (2021). Effects of dietary protein levels on growth performance, digestibility, anti-oxidative responses and expressions of growth-related genes in triploid rainbow trout *Oncorhynchus mykiss* farmed in seawater. *Aquaculture Nutrition*.

[29] Blaxter K. L. (1990). Energy metabolism in animals and man. *Animal Behaviour*.

[30] AOAC (Association of Official Analytical Chemists) (2005). *Official Methods of Official Analytical Chemists International*.

[31] Yang H., Li X.-Q., Xu Z., Cheng Z., Leng X.-J. (2020). Effects of three active components in *Eucommia ulmoides* on growth and flesh quality of grass carp (*Ctenopharyngodon idellus*) based on transcriptomics. *Aquaculture Nutrition*.

[32] Norambuena C., Mussa K., Hernández F., Alfaro J., Velasco M. (2019). Energy balance of pregnant vicuñas (*Vicugna vicugna*) in the Chilean High Andes. *Austral Journal of Veterinary Sciences*.

[33] Mohanta K. N., Mohanty S. N., Jena J., Sahu N. P. (2009). A dietary energy level of 14.6 MJ kg^−1^ and protein-to-energy ratio of 20.2 g MJ^−1^ results in best growth performance and nutrient accretion in silver barb *Puntius gonionotus* fingerlings. *Aquaculture Nutrition*.

[34] Zhang J., Li Y., Sun G., Zhao N., Ma J., Wang S. (2017). Effects of lipid levels on growth, fatty acid enzymatic activity, and gene expression of rainbow trout (*Oncorhynchus mykiss*) in a recirculating aquaculture system. *Marine Sciences*.

[35] Imsland A. K., Foss A., Folkvord A., Stefansson S. O., Jonassen T. M. (2005). The interrelation between temperature regimes and fish size in juvenile Atlantic cod (*Gadus morhua*): effects on growth and feed conversion efficiency. *Fish Physiology and Biochemistry*.

[36] Hidalgo F., Alliot E., Thebault H. (1987). Influence of water temperature on food intake, food efficiency and gross composition of juvenile sea bass, *Dicentrarchus labrax*. *Aquaculture*.

[37] Mishra K., Samantaray K. (2004). Interacting effects of dietary lipid level and temperature on growth, body composition and fatty acid profile of rohu, *Labeo rohita* (Hamilton). *Aquaculture Nutrition*.

[38] Garcia M., Zamora S., Lopez M. A. (1981). The influence of partial replacement of protein by fat in the diet on protein utilization by the rainbow trout (*Salmo gairdneri*). *Comparative Biochemistry and Physiology Part B: Comparative Biochemistry*.

[39] Khan S., Ang K. J., Ambak M. A. (1996). The effect of varying dietary protein level on the growth, food conversion, protein utilization and body composition of tropical catfish *Mystus nemurus* (C. & V.) cultured in static pond water systems. *Aquaculture Research*.

[40] Du Z.-Y., Liu Y.-J., Tian L.-X., Wang J.-T., Wang Y., Liang G.-Y. (2005). Effect of dietary lipid level on growth, feed utilization and body composition by juvenile grass carp (*Ctenopharyngodon idella*). *Aquaculture Nutrition*.

[41] Guo J.-L., Zhou Y.-L., Zhao H., Chen W.-Y., Chen Y.-J., Lin S.-M. (2019). Effect of dietary lipid level on growth, lipid metabolism and oxidative status of largemouth bass, *Micropterus salmoides*. *Aquaculture*.

[42] Alanärä A., Kiessling A. (1996). Changes in demand feeding behaviour in Arctic charr, *Salvelinus alpinus* L., caused by differences in dietary energy content and reward level. *Aquaculture Research*.

[43] Koskela J., Jobling M., Savolainen R. (1998). Influence of dietary fat level on feed intake, growth and fat deposition in the whitefish *Coregonus*. *Aquaculture International*.

[44] Wang J., Liu T., Zheng P. (2021). Effect of dietary lipid levels on growth performance, body composition, and feed utilization of juvenile spotted knifejaw *Oplegnathus punctatus*. *Aquaculture Reports*.

[45] Peres H., Oliva-Teles A. (1999). Effect of dietary lipid level on growth performance and feed utilization by European sea bass juveniles (*Dicentrarchus labrax*). *Aquaculture*.

[46] Xu X., Yang H., Zhang C. (2022). Effects of replacing fishmeal with cottonseed protein concentrate on growth performance, flesh quality and gossypol deposition of largemouth bass (*Micropterus salmoides*). *Aquaculture*.

[47] Gylfason G. A., Knútsdóttir E., Ásgeirsson B. (2010). Isolation and biochemical characterisation of lipid rafts from Atlantic cod (*Gadus morhua*) intestinal enterocytes. *Comparative Biochemistry and Physiology Part B: Biochemistry and Molecular Biology*.

[48] Calder P. C. (2015). Functional roles of fatty acids and their effects on human health. *Journal of Parenteral and Enteral Nutrition*.

[49] Borges P., Oliveira B., Casal S., Dias J., Conceição L., Valente L. M. P. (2009). Dietary lipid level affects growth performance and nutrient utilisation of Senegalese sole (*Solea senegalensis*) juveniles. *British Journal of Nutrition*.

[50] Li F.-J., Lin X., Lin S.-M., Chen W.-Y., Guan Y. (2016). Effects of dietary fish oil substitution with linseed oil on growth, muscle fatty acid and metabolism of tilapia (*Oreochromis niloticus*). *Aquaculture Nutrition*.

[51] Madsen L., Froyland L., Dyroy E., Helland K., Berge R. K. (1998). Docosahexaenoic and eicosapentaenoic acids are differently metabolized in rat liver during mitochondria and peroxisome proliferation. *Journal of Lipid Research*.

[52] Ren Y., Wei S., Yu H. (2021). Dietary lipid levels affect growth, feed utilization, lipid deposition, health status and digestive enzyme activities of juvenile Siberian sturgeon, *Acipenser baerii*. *Aquaculture Nutrition*.

[53] Guo Z., Zhu X., Liu J. (2011). Dietary lipid requirement of juvenile hybrid sturgeon, *Acipenser baerii*♀×*A. gueldenstaedtii*♂. *Journal of Applied Ichthyology*.

[54] Babin P. J., Vernier J. M. (1989). Plasma lipoproteins in fish. *Journal of Lipid Research*.

[55] Dias J., Alvarez M. J., Diez A. (1998). Regulation of hepatic lipogenesis by dietary protein/energy in juvenile European seabass (*Dicentrarchus labrax*). *Aquaculture*.

[56] Moon T. W. (1988). Adaptation, constraint, and the function of the gluconeogenic pathway. *Canadian Journal of Zoology*.

[57] Zhao P.-F., Li F.-J., Chen X.-R. (2016). Dietary lipid concentrations influence growth, liver oxidative stress, and serum metabolites of juvenile hybrid snakehead (*Channa argus* × *Channa maculata*). *Aquaculture International*.

[58] Grassmann E., Tschierschwitz A., Roth F. X., Kirchgessner M. (2009). Protein supply of rats and transaminase and succinate dehydrogenase activity. *Archiv für Tierernaehrung*.

[59] Kim K.-I., Grimshaw T. W., Kayes T. B., Amundson C. H. (1992). Effect of fasting or feeding diets containing different levels of protein or amino acids on the activities of the liver amino acid-degrading enzymes and amino acid oxidation in rainbow trout (*Oncorhynchus mykiss*). *Aquaculture*.

[60] SÁ R., Pousão-Ferreira P., Oliva-Teles A. (2008). Dietary protein requirement of white sea bream (*Diplodus sargus*) juveniles. *Aquaculture Nutrition*.

[61] Siddiqua K. S., Khan M. A. (2022). Effects of dietary lipid levels on growth, feed utilization, RNA/DNA ratio, digestive tract enzyme activity, non-specific immune response and optimum inclusion in feeds for fingerlings of rohu, *Labeo rohita* (Hamilton). *Aquaculture*.

[62] Wang C., Hu G., Sun P. (2018). Effects of dietary protein at two lipid levels on growth, gonadal development, body composition and liver metabolic enzymes of brown trout (*Salmo trutta fario*) broodstock. *Aquaculture Nutrition*.

